# Effect of Pooling and Freeze-Thaw Processes on the PRRSV RNA Detection in TOSc Samples

**DOI:** 10.3390/vetsci12070607

**Published:** 2025-06-21

**Authors:** Peng Li, Onyekachukwu H. Osemeke, Thinh Tran Pham Tien, Ching-Yang Wang, Angie Supple, Marcelo N. Almeida, Daniel C. L. Linhares

**Affiliations:** 1 College of Veterinary Medicine, Iowa State University, Ames, IA 50010, USA; lipeng@iastate.edu (P.L.);; 2 Eichelberger Farms, Wayland, IA 52654, USA

**Keywords:** PRRSV detection, pooling, freeze-thaw, TOSc

## Abstract

Tonsil oral scrubbing (TOSc) is a simple and practical method recently developed for detecting PRRSV RNA in sows. This study evaluated how pooling and freeze-thaw procedures affect PRRSV RT-rtPCR results in TOSc samples. To assess pooling effects, 22 PRRSV-positive samples were divided into three Ct categories and diluted with negative TOSc samples at various ratios before testing. To assess freeze-thaw effects, 90 TOSc samples were split into two equal aliquots: one tested immediately, and the other tested after one freeze-thaw cycle. Results showed that PRRSV RNA detection probability decreased and Ct values increased as dilution (pooling) ratios rose. Samples with Ct values > 35 had significant drops in probability of detection beginning at a 1:3 dilution (≤71%). In contrast, samples with Ct values <35 maintained a high probability of detection until 1:10 dilution (≥85%). The freeze-thawed samples showed significantly higher Ct values and numerically lower probability of detection compared to non-freeze-thawed ones. In conclusion, pooling and freeze-thaw negatively impact PRRSV RNA detection in TOSc samples. Pooling may be acceptable for samples with a Ct value < 35 for up to 1:10 dilution, but should be avoided when the Ct value > 35. Similarly, the freeze-thaw process should be minimized during TOSc sample handling.

## Simple Summary

Tonsil oral scrubbing (TOSc) is a recently developed, easy, and practical sampling method for detecting PRRSV RNA in breeding sows. This study aimed to evaluate whether pooling samples or subjecting them to a freeze-thaw cycle would affect PRRSV RNA detection. We tested pooled samples at various dilution ratios and compared PRRSV RNA detection in fresh versus freeze-thawed TOSc samples. Results showed that pooling reduced probability of PRRSV RNA detection, especially in samples with Ct values > 35, while those with Ct values < 35 remained detectable even after pooling to a ratio of 1:10. Similarly, one freeze-thaw cycle increased Ct values and reduced probability of detection. These findings suggest that pooling may be used in TOSc samples with low Ct values < 35 to reduce testing costs, but should be avoided for samples with Ct values > 35. Freeze- thawed samples should also be minimized during handling. This research provides practical TOSc sample handling guidance for laboratories and veterinarians to optimize PRRSV surveillance using TOSc.

## 1. Introduction

As sows are a major source of porcine reproductive and respiratory syndrome virus (PRRSV) [1], monitoring sows for PRRSV infection is important to reveal the level of virus endemicity in each herd. A novel sow sampling method, tonsil oral scrubbing (TOSc), was developed by recovering fluids from the sow tonsillar area and the oral cavity without restraining the sows [2,3,4,5]. TOSc samples had a similar decreasing trend of PRRSV RNA detection with tonsil scraping samples over time, having higher positivity than that of oral fluid and serum [4]. Moreover, TOSc samples could be collected within 20 s [2], making possible an easy collection of a large amount of sow samples and investigation of PRRSV dynamics in sows especially when reaching low prevalence. However, in the field, the budget for testing is often restricted.

Pooling is a practical strategy to reduce diagnostic cost for nucleic acid detection in different sample types, especially when dealing with a large number of samples [6,7,8,9]. It was described that pooling of family oral fluid (FOF) at dilution level of 1:20 did not significantly change the probability of PRRSV RNA detection [10]. Even though the effect of diluting samples on the probability of analyte detection is theoretically predictable, the extent of changes can vary among sample types and varying field conditions [6,7,8,11]. Thus, it is worthwhile investigating the effect of pooling on the PRRSV RNA detection for TOSc samples.

Freeze-thaw is a common step in the process of viral isolation and detection by breaking down cell components and releasing viral particles and/or nucleic acid [6,7,12]. TOSc samples include biological materials from the tonsillar area; clusters of immune cells including macrophages were revealed in TOSc samples by histopathology [5], and PRRSV mainly replicates in macrophages [13,14,15,16]. Thus, it is also crucial to investigate the effect of freeze-thaw on PRRSV RNA detection in an attempt to refine the testing procedure for TOSc samples.

In summary, the overall objective of this study is to evaluate the effect of pooling and freeze-thaw on the probability of PRRSV RNA detection and Ct values by Real-Time reverse transcription–Polymerase Chain Reaction (RT-rtPCR) in TOSc samples.

## 2. Materials and Methods

### 2.1. Study Design

To test the effect of pooling on detection for PRRSV RNA, 22 known RT-rtPCR-positive TOSc samples from PRRSV endemic herds were categorized into 3 groups by Ct values: Category A, 29 < Ct ≤ 32, *n* = 7; Category B, 32 < Ct ≤ 35, *n* = 9; Category C, 35 < Ct ≤ 38, *n* = 6. Each sample was diluted with pooled TOSc samples known to be RT-rtPCR-negative from naïve herds at different ratios: 1:3, 1:5, 1:8, and 1:10, respectively. For example, at a ratio of 1:3, 333 µL of positive samples were diluted by 667 µL of negative samples. Likewise, 200, 125, and 100 µL of positive samples were diluted by 800, 875, and 900 µL of negative samples at ratios of 1:5, 1:8, and 1:10, respectively. Each original and pooled sample was tested by PRRSV RT-rtPCR with 6 replicates.

To test the freeze-thaw effect on PRRSV RNA detection, 90 TOSc samples were conveniently collected from PRRSV-endemic herds. Each sample was mildly vortexed and equally aliquoted into 2 replicates after arrival at the laboratory. One aliquot was submitted for testing for PRRSV by RT-rtPCR (non-freeze-thaw group), while the other aliquot was frozen at −20 °C overnight and thawed at −4 °C for 8 h (freeze-thaw group) and then submitted for testing using the same protocols and conditions.

The Institutional Animal Care and Use Committee (IACUC) of Iowa State University, IA, approved this study (IACUC-22-101).

### 2.2. Sample Collection

TOSc Samples Collection

TOSc samples were collected without restraining the sows as previously described [2,3]. The head of the artificial insemination rod was placed beside the mouth of the sow to attract her attention. The collector was then inserted into the sow’s mouth, directed toward the tonsillar area with an upwards angle, and then scrubbed back and forth for ten seconds. The qualified sample was viscous and mucous-like. The head part of the rod with the sample on it was cut by a sterilized clip and then transferred to a 50 mL conical tube (Corning Science Mexico S.A. de C.V., Tamaulipas, Mexico) with three ml of PBS.

### 2.3. Diagnostic Testing

All samples were individually tested for PRRSV RNA by RT-qPCR using validated protocols at Iowa State University Veterinary Diagnostic Laboratory (ISU-VDL). Ct values of less than 40 were considered positive [2,4].

### 2.4. Sample Size Justification

To assess the effect of pooling, a sample size of 36 (6 replicates of 6 samples per group) per dilution group was estimated to detect a 25% difference in the probability of detection with a significance level (α) of 5% and a power of 80%.

To assess the effect of freeze-thaw, a sample size of 90 per group was justified to detect a 15% difference in the probability of detection with a significance level (α) of 5% and a power of 80%.

### 2.5. Statistical Analysis

A probit regression model using the brglm function (Bias Reduction in Binomial-Response Generalized Linear Models) was used to define the probability of detection (Ppcr) for each category with a binary outcome (RT-rtPCR-positive or -negative) as the response variable and dilution level as the predictor (categorical) variable [10,17]:

(1) $$\Phi^{-1}\left(\hat{P}pcr\right)=\alpha +\beta x\;$$

A generalized linear mixed model was used to model the logit of detection rate as a function of freeze-thaw, and the difference in probability of detection between freeze-thaw group and non-treatment group was assessed. In this model, the outcome (RT-rtPCR-positive or -negative) was the dependent variable, freeze-thaw was the fixed effect, with sample identification number as a random effect. Specifically, the Tukey–Kramer test was used as a post hoc test to compare the marginal means of detection rates. The signed-rank test was used to compare differences between Ct values of the paired freeze-thaw group and non-treatment group [18]. All analyses were performed using R version 4.4.2 (R Core Team, 2019).

## 3. Results

### 3.1. Ct Value Changes per Dilution Level for Each Category

**Figure 1:**
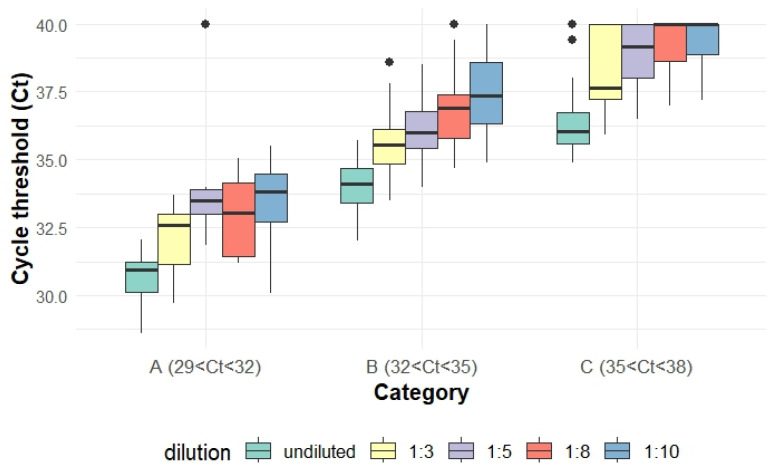
Box plot depicting Ct value variations across different dilution levels within each Ct value category. 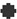 the dots means data points falling outside of whiskers, usually 1.5× the interquartile range, IQR.

The Ct values increased progressively with higher dilution levels across all categories. The range of mean Ct value changes was 1.4–1.8, 2.2–2.6, 2.4–3.0, and 2.8–3.6 for dilution levels of 1:3, 1:5, 1:8, and 1:10, compared with undiluted groups, respectively (Figure 1, Table 1).

### 3.2. Changes of Probability of PRRSV RNA Detection After Different Levels of Dilution for Each Ct Value Category

**Figure 2:**
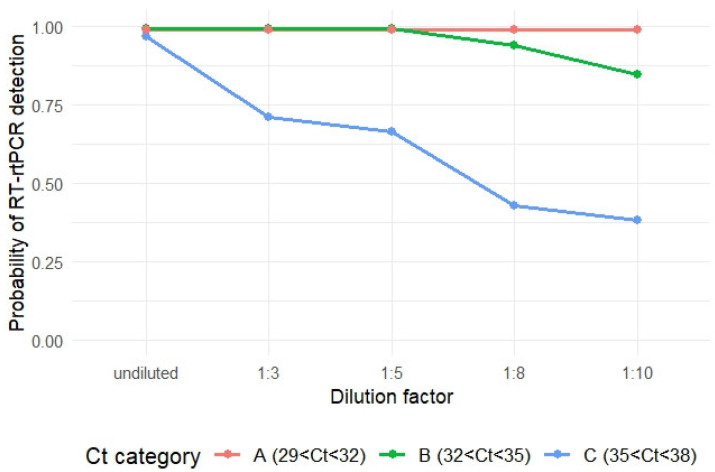
Changes of probability of PRRSV RNA detection after different levels of dilution for each Ct value category.

The mean probability of PRRSV RNA detection for Category A (29 < Ct < 32) remained 99% after all levels of dilution, while that for Category B (32 < Ct < 35) decreased to 95%, and 84% at dilution ratio of 1:8 and 1:10, respectively, compared to undiluted groups. However, the decrease in probability of PRRSV RNA detection was not statistically significant (Tukey test, *p* > 0.05). For Category C (35 < Ct < 38), the probability of PRRSV RNA detection significantly decreased at all dilution levels compared to undiluted samples (Tukey test, *p* < 0.05). The mean detection probabilities were 71% (1:3), 66% (1:5), 43% (1:8), and 38% (1:10), compared to 97% for undiluted samples (Table 2 and Figure 2).

### 3.3. Comparison of Probability of PRRSV RNA Detection and Difference in Ct Values Between Non-Freeze-Thaw and Freeze-Thaw Groups

While the non-freeze-thaw group showed a numerically higher probability of PRRSV RNA detection (0.32; 95% confidence interval, 0.23–0.42) than the freeze-thaw group (0.28; 95% confidence interval, 0.15–0.46), the difference was not statistically significant (Tukey test, *p* = 0.12). However, the non-freeze-thaw group showed significantly lower Ct values than the freeze-thaw group with a mean difference of 0.24 (signed-rank test, *p* < 0.001) (Table 3).

## 4. Discussion

The objective of this study was to assess the pooling and freeze-thaw effects on the probability of PRRSV RNA detection and Ct values for TOSc samples. Pooling is a cost-effective method to detect pathogens, especially when the budget for testing is restrained. Pooling effect was previously evaluated on multiple sample types, including oral fluid (OF) [19], processing fluid (PF) [8], and FOF [10] to detect swine pathogens by PCR-based assays. The increase in cycle threshold (Ct) values at different dilution levels in a RT-rtPCR assay is determined by the logarithmic nature of PCR amplification. Theoretically, for a perfect qPCR reaction with 100% efficiency, a 10-fold dilution (1:10) leads to an increase of approximately 3.32 Ct values because ΔCt = log_2_(Dilution Factor) [20,21]. Using this formula, the theoretical increase in Ct values for each dilution level will be 1.58, 2.32, and 3 and 3.32 at dilution factors of 1:3, 1:5, 1:8, and 1:10, respectively. In this study, the ranges of mean Ct value changes for each category were 1.4–1.8, 2.2–2.6, 2.4–3.0, and 2.8–3.6 after each dilution level, respectively. The range centered around the theoretical value and was within expectation as in real-world experiments, the actual Ct value shifts might be slightly different due to RT-rtPCR efficiency deviations (e.g., inhibitors, pipetting errors, or suboptimal reagent conditions) [22,23,24,25]. This was also consistent with previous research reporting an average increase of 1.28, 2.09, 2.90, and 4.12 in Ct values in FOF stock samples when diluting at 1:3, 1:5, 1:10, and 1: 20 levels, respectively [10].

Ideally, Equation (1) would be a mixed probit model with individual samples used as random effects. However, the model would not converge by incorporating random effects in the context of perfect separation as all undiluted replicates across all samples were always PRRSV RT-rtPCR-positive. Thus, a bias reduction model was employed [10,17].

For both A and B categories, the probability of PRRSV RNA detection did not change significantly for all dilution levels. Thus, pooling of TOSc samples up to 1:10 dilution level would not affect the probability of PRRSV RNA detection for samples with Ct values of less than 35. However, for Category C (35 < Ct < 38), there were significant drops in probability of detection since a dilution ratio of 1:3 compared with undiluted samples (Tukey test, *p* < 0.05). This was possibly attributed to the fact that PRRSV RNA in samples from Category C was close to the detection limit and each dilution pushes several samples beyond the limit of detection, where stochastic effects and low template copies result in a high rate of false negatives [20,26,27,28]. This drop in probability of detection after dilution of high Ct value samples was also observed in another study evaluating the pooling effect on PRRSV RNA detection in FOF samples [10].

However, in contrast to the Ct value distribution of FOF samples with a mean Ct value of 31.48 [10], that of TOSc samples was right-skewed with a mean Ct value of 33.5. Moreover, around 25% of the positive TOSc samples submitted to the ISU Veterinary Diagnostic Lab fell into the Ct range of 35–38. This raised a particular concern about using a pooling strategy in an attempt to detect positive sows by TOSc samples, especially at low PRRSV prevalence, where there are fewer positive sows and with higher Ct values. Pooling at a ratio of 1:3 will lead to a drop in the mean probability of PRRSV RNA detection to 71%. On the other hand, pooling increases the probability of including more positive animals, and the current probit regression model evaluated a “worst-case scenario” in which TOSc samples were combined with known PRRSV-negative TOSc samples. Thus, pooling per se may lead to diluting the sample. However, using pooling to increase the coverage of the animals sampled in the population leads to an increase in the overall probability of RNA detection. Another key consideration in determining whether to adopt a pooling strategy for TOSc samples at low prevalence is the objective of sample collection and the difference between the interpretation of TOSc and FOF samples. While FOF samples are primarily collected from litters over 16 days of age, they reflect the PRRSV status of the weaning-age piglet population [10], where maternally derived immunity gradually declines over time [29,30,31,32,33]. A positive FOF sample may therefore be linked to poorer performance in the grow-finish phase [34,35]. In contrast, TOSc samples are taken from sows with existing immunity, meaning that failing to detect a positive sow with Ct value of 35 or 38 from TOSc samples may not necessarily indicate a significant production impact on the breeding herd. However, this warrants further investigation in future studies. To sum up, caution is needed when pooling for TOSc samples with Ct value larger than 35.

The effects of temperature and time and freeze-thaw have been described on PRRSV RNA detection in OF samples [6]. Due to the presence of small amounts of clustered suspect macrophages in TOSc samples [5], we hypothesized that the freeze-thaw process, which is a common step in viral detection process [6,7,12], would release the PRRSV RNA from those macrophages and increase PRRSV RNA detection rate and decrease Ct values from TOSc samples. One cycle of freeze-thaw treatment with freezing at −20 °C overnight and thawing at 4 °C for 8 h was conducted in this study to simulate the common practice of storing (−20 °C overnight) and delivery (0–4 °C on ice) process for a common sample type in the farm. However, the results showed that the non-freeze-thaw group had numerically higher probability of detection (Tukey test, *p* = 0.12) and significantly lower mean Ct values (signed-rank test, *p* < 0.001) than the freeze-thaw groups. This was possibly attributable to the fact that during the freeze-thaw cycle, PRRSV RNA was degraded [6,12]. On average, the mean Ct value increased by 0.24 in TOSc samples following a freeze-thaw cycle, aligning with a previous study that reported a 0.03 Ct increase in OF samples after a similar freeze-thaw process (frozen at −80 °C in the morning and thawed at 4 °C overnight) [6]. While a positive effect of freeze-thaw on RNA release into the supernatant cannot be ruled out in this study, it clearly demonstrates the negative impact of RNA degradation. Therefore, for TOSc sample handling in the field, freeze-thaw cycles should be avoided to prevent RNA degradation and ensure optimal PRRSV RNA detection.

## 5. Conclusions

The probability of PRRSV RNA detection decreased and Ct values increased progressively in TOSc samples with increasing dilutions, and one cycle of freeze-thaw process decreased probability of PRRSV RNA detection and increased Ct values in TOSc samples. Pooling may be acceptable for samples with Ct value < 35 for up to 1:10 dilution, but should be avoided when Ct value > 35. Similarly, the freeze-thaw process should be minimized during TOSc sample handling.

## Figures and Tables

**Table 1:** Volumes of PRRSV-positive and PRRSV-negative TOSc samples.

Dilution Level	Undiluted	1:3	1:5	1:8	1:10
Category A (29 < Ct ≤ 32)	30.6	32.0 (1.4)	33.2(2.6)	33.0 (2.4)	33.4 (2.8)
Category B (32 < Ct ≤ 35)	33.9	35.5 (1.6)	36.1 (2.2)	36.8 (2.9)	37.5 (3.6)
Category C (35 < Ct ≤ 38)	36.3	38.1 (1.8)	38.9 (2.6)	39.3 (3.0)	39.4 (3.1)

**Table 2:** Changes in the probability of PRRSV RNA detection after different levels of dilution for each Ct value category.

Pooling Level	Mean (95% Confidence Interval)		
	Category A: 29 < Ct < 32	Category B: 32 < Ct < 35	Category C: 35 < Ct < 38
Undiluted	0.99 (0.77–1.00) ^a^	0.99 (0.84–1.00) ^a^	0.97 (0.81–0.99) ^c^
1:3	0.99 (0.77–1.00) ^a^	0.99 (0.84–1.00) ^a^	0.71 (0.51–0.86) ^b^
1:5	0.99 (0.77–1.00) ^a^	0.99 (0.84–1.00) ^a^	0.66 (0.46–0.83) ^ab^
1:8	0.99 (0.77–1.00) ^a^	0.94 (0.80–0.99) ^a^	0.43 (0.25–0.63) ^ab^
1:10	0.99 (0.77–1.00) ^a^	0.85 (0.69–0.94) ^a^	0.38 (0.21–0.58) ^a^

^a,b,c^: different letters within columns indicate statistical difference (*p* < 0.05, Tukey test).

**Table 3:** Comparison of the probability of PRRSV RNA detection and the difference in Ct values between non-freeze-thaw and freeze-thaw groups.

Dilution Level	Non-Freeze-Thaw	Freeze-Thaw
Probability of detection with 95% CI	0.32 (0.23–0.43%) ^a^	0.28 (0.15–0.46) ^a^
Difference in Ct values in paired samples	Δ = 0.24 *	

^a^: non-significant difference on the probability of PRRSV RNA detection (Tukey test, *p* = 0.12) between groups. Δ: mean difference in Ct values between two groups. *: statistically significant difference between two groups (signed-rank test, *p* < 0.001).

## Data Availability

Data is contained within the article.

## Abbreviations

The following abbreviations are used in this manuscript:

PRRSV	Porcine reproductive and respiratory syndrome virus
TOSc	Tonsil oral scrubbing
